# Trends in HIV Testing Among Adults in the Deep South: Behavioral Risk Factor Surveillance System, 2017–2023

**DOI:** 10.1007/s10461-025-04776-x

**Published:** 2025-06-17

**Authors:** Precious Patrick Edet, Azad R. Bhuiyan, Trisha Arnold, Amy Nunn, Andrew Yockey, Ruaa Al Juboori, Hannah K. Allen

**Affiliations:** 1 Department of Public Health, University of Mississippi, University, MS 38677 USA; 2 William Magee Institute for Student Wellbeing, University of Mississippi, University, MS 38677 USA; 3 Department of Epidemiology and Biostatistics, Jackson State University, Jackson, MS 39213 USA; 4 Department of Psychiatry and Human Behavior, Brown University Health, Providence, RI 02912 USA; 5 Department of Behavioral and Social Health Sciences, Brown University, Providence, RI 02912 USA

**Keywords:** HIV testing, Deep south, BRFSS, Trend analysis

## Abstract

HIV testing is an entry point for both HIV prevention and treatment, and the CDC recommends that all adults of reproductive age undergo HIV testing at least once in their lifetime. However, HIV testing rates remain suboptimal. This study analyzed trends in HIV testing using Behavioral Risk Factor Surveillance System data from 2017 to 2023 across nine Deep South states—Alabama, Florida, Georgia, Louisiana, Mississippi, North Carolina, South Carolina, Tennessee, and Texas. Descriptive statistics and Joinpoint linear regression were employed to assess lifetime HIV testing and testing within the past 12 months. Findings showed that the overall trend in having ever tested for HIV rose from 43% in 2017 to 47% in 2019 but declined to 40% in 2022, with a slight increase to 41% in 2023. Significant declines in ever testing were observed in North Carolina and among adults aged 25–44, non-Hispanic Black, non-Hispanic multiracial, and those identifying as lesbian or gay. Furthermore, the overall trend in HIV testing in the past 12 months declined significantly from 48% in 2017 to 42% in 2022, with a slight increase to 43% in 2023. Significant declines were found in Florida, Georgia, Louisiana, North Carolina, and South Carolina, and among adults aged 18–34, non-Hispanic White, non-Hispanic Black, male, female, and heterosexual individuals. These trends, observed largely during the COVID-19 pandemic, underscore the need to scale up HIV prevention and care initiatives, particularly in populations and regions experiencing significant declines. Trends should continue to be monitored and examined post-COVID pandemic.

## Introduction

In 2022, approximately 1.2 million people aged 13 or older were living with HIV knowingly or unknowingly in the United States (U.S.) [1, 2]. Despite a 12% decrease in new HIV infections from 2018 to 2022, more than 38,000 people received an HIV diagnosis in 2022 [1, 3, 4]. The Southern region of the United States has consistently faced the highest HIV burden, accounting for more than half (51–53%) of all HIV infections in the U.S [3, 5–7]. Within this region, the Deep South—Alabama, Florida, Georgia, Louisiana, Mississippi, North Carolina, South Carolina, Tennessee, and Texas, has been particularly impacted, with an HIV diagnosis rate as high as 65% [8].

The federal initiative “Ending the HIV Epidemic in the U.S.” (EHE) by the U.S. Department of Health and Human Services (DHHS) aims to reduce new HIV infections in the United States by 75% and 90% by 2025 and 2030, respectively [9]. Additionally, Healthy People 2030 goals set by the Office of Disease Prevention and Health Promotion within the DHHS emphasizes increasing the proportion of people who are aware of their HIV status through testing and reducing the number of new HIV infections by 2030 [10].

HIV testing is the entryway for HIV prevention and treatment [11]. The Centers for Disease Control and Prevention (CDC) report and several studies have confirmed that timely HIV testing and treatment reduce transmission, particularly when combined with strategies like pre-exposure prophylaxis (PrEP) [12–14]. The CDC recommends that all individuals aged 13 to 64 years should get tested for HIV at least once in their lifetime, and at-risk individuals, including racial and sexual minorities and people who inject drugs, should get tested more often—at least once a year [12, 15]. However, despite these recommendations and ongoing prevention efforts, HIV testing rates are suboptimal, particularly among people of color (57–60%) who are at highest risk of HIV acquisition [11, 16–19].

Several studies have investigated HIV testing trends across the U.S. over time. Ansa and colleagues [20] used statewide data for Georgia and revealed a slight, although not significant, decline in ever testing for HIV (46% in 2011 to 44% in 2015). Moreover, another study by Patel and colleagues [21] demonstrated a significant increase in ever testing for HIV (43% in 2011 to 46% in 2017), and the rate of testing within the past 12 months increased from 13 to 15% during the same period. Despite these upward trends, Patel et al. [21] highlighted that fewer than half of U.S. adults had ever tested for HIV during the study period, even after a decade since CDC’s recommendations on HIV testing were published. Additionally, the CDC [4] reported an increase in HIV testing across the U.S. in 2022, adding that a higher percentage of people with HIV were aware of their status in 2022 than in 2018, with a slight increase from 86 to 87%.

The Southern U.S. is identified as a priority region in the EHE plan, given that it accounts for more than half of all new HIV diagnoses nationally [3, 5–7]. However, the Deep South continues to have poorer HIV outcomes compared to other Southern States [8, 22], with 20 counties from this region listed as priority areas in the EHE plan [9]. According to Reif et al. [23], the rate of HIV diagnoses in the Deep South states was 24 per 100,000 people in 2014, which was 45% higher than the rate in other Southern states (Arkansas, Delaware, District of Columbia, Kentucky, Maryland, Oklahoma, Virginia, and West Virginia), which had a combined diagnosis rate of 17 per 100,000 people, aligning with the national average. Examining trends in HIV testing in the Deep South is critical for ultimately reducing transmission rates.

This study had two aims: (1) examine trends in ever been tested for HIV (i.e., a person has ever been tested at some time in their life) among adults in the Deep South; and (2) examine HIV testing in the past 12 months among adults in the Deep South, utilizing the 2017–2023 Behavioral Risk Factor Surveillance System (BRFSS) data. This study will identify specific demographic groups with potential recent declines in HIV testing who may benefit from targeted intervention efforts. Findings from this study will also contribute to monitoring progress toward achieving national EHE and Healthy People 2030 goals and may help inform public health interventions, thereby contributing to the broader goal of preventing HIV spread and ending the HIV epidemic in the United States.

## Methods

### Study Design and Participants

This study utilizes cross-sectional study data from the 2017*–*2023 BRFSS to investigate trends in ever been tested for HIV and HIV testing in the past 12 months in the Deep South region of the United States—Alabama [AL], Florida [FL], Georgia [GA], Louisiana [LA], Mississippi [MS], North Carolina [NC], South Carolina [SC], Tennessee [TN], and Texas [TX], as defined in previous studies [8, 23]. While each year’s data represents a cross-sectional snapshot, the multi-year analysis allows for the assessment of HIV testing trends over time. Participants were at least 18 years old, non-institutionalized, resided in the Deep South, and had access to either a landline or cell phone. The state of Florida did not collect BRFSS data in 2021.

### Data Source

This study utilized secondary data from the 2017–2023 BRFSS datasets with selected cases from states in the Deep South. The BRFSS dataset is a nationally representative dataset administered annually by the CDC across all 50 U.S. states, District of Columbia, and U.S. territories including Guam, Puerto Rico, and the Virgin Islands [24]. The BRFSS dataset utilizes a complex sampling methodology to ensure that data collected is generalizable to the general population [24]. To accommodate the complex sampling design, the BRFSS data was weighted, clustered, and stratified so that findings are generalizable to the entire population. The BRFSS collects data on health risk behaviors, preventive services and chronic health conditions from a representative sample of noninstitutionalized adults aged 18 years or older residing in the United States and territories [24]. Data were collected through landline and cellular telephones by means of a Random-Digit Dialing [24].

Weighting procedures employed by BRFSS changed in 2011 when it began using a new methodology known as iterative proportional fitting, also known as “raking” [24]. This process utilized data from cellular telephone surveys allowing for the addition of three demographic characteristics (education, marital status, home renter/owner) to age, race/ethnicity, and sex, to reduce biases associated with low survey coverage of people with certain demographic characteristics [24]. These additional characteristics made the BRFSS sample more representative and generalizable with respect to individual states’ populations based on the largest possible cross-section of demographic characteristics [24].

### Measures

#### Independent Variables

The independent variables were sociodemographic factors including state (AL, FL, GA, LA, MS, NC, SC, TN, and TX), age group (18–24, 25–34, 35–44, 45–54, 55–64, and 65 or older), race/ethnicity (non-Hispanic [NH] White, NH Black, NH other race, NH multiracial, and Hispanic), sex (male and female), sexual orientation (lesbian or gay, heterosexual, bisexual, and other), education level (less than high school, high school, some college, and college graduate), annual income level in U.S. Dollars (<$15k, $15k–<$25k, $25k–<$35k, $35k–<$50k, and $50k or more), employment status (employed for wages, self-employed, unemployed, and retired), and marital status (not married, married, divorced, and widowed), which provided information on the demographic characteristics of the study population. These variables were also used to estimate the percentage of ever been tested for HIV and testing for HIV in the past 12 months among adults in the Deep South.

#### Dependent Variables

The dependent variables were HIV testing (ever) and HIV testing in the past 12 months. To obtain data on ever testing for HIV, participants were asked, “Have you ever been tested for HIV?” Responses included “yes”, “no”, “don’t know” and “refused”. To obtain data on HIV testing in the past 12 months, only participants who reported ever testing for HIV were asked, “In what month and year was your last HIV test?” HIV tests conducted at any time in the preceding year beginning in January (12 months from the start of each interview year) through December of the current interview year (end of each interview year) were coded as tests conducted in the past 12 months from the date of interview in each given year. Responses were then dichotomized into two categories: “last HIV testing done in the past 12 months” and “last HIV testing done in more than 12 months.”

All “don’t know,” “refused,” and missing responses were excluded from the analysis to minimize the underestimation of HIV testing and data accuracy, and to be consistent with similar prior analyses [20, 21, 25–27].

### Data Analysis

Prior to analyzing the data, the BRFSS dataset was weighted, clustered, and stratified using _LLWPWT (Final Weight), _PSU (Primary Sampling Unit), and _STRTR (Stratification) variables, respectively, to ensure that the data was accurately analyzed so findings were generalizable to the target population. Descriptive statistics of dependent and independent variables were then performed for each year using Proc surveyfreq procedures in SAS. Weighted frequencies, unweighted count, and 95% confidence intervals (95% CI) provided descriptive statistical information about the study population.

To examine trends, we used descriptive statistics to calculate estimated percentages and their respective standard errors of ever been tested for HIV and testing for HIV in the past 12 months among adults in the Deep South, overall, and by state, age group, race/ethnicity, sex, sexual orientation, education level, annual income level (USD), employment status, and marital status, using Proc surveyfreq procedures in SAS.

Additionally, we performed Joinpoint linear regression to determine the Annual Percent Change (APC) and Average Annual Percent Change (AAPC) for each trend observed during the study period. An Excel spreadsheet comprising each given year of interview, estimated weighted percentages of ever been tested for HIV or HIV testing in the past 12 months by sociodemographic factors for the corresponding year, and standard errors of weighted percentages were used to create the Joinpoint input file which was then uploaded to the Joinpoint software. Permutation test was employed to identify the number of join points, while a parametric method was utilized to calculate the 95% CI for the APC and AAPC for overall trends [28]. Significant changes were identified as shifts in the rate of increase or decrease in the AAPC, as indicated by a 95% CI which did not include 1. The assumptions for Joinpoint linear regression were assessed and validated, ensuring the accuracy of the results.

SAS Institute, Incorporated, statistical package version 9.4 [29] and Joinpoint software version 5.1.0 (Available online: https://surveillance.cancer.gov/joinpoint/) [28, 30] were used to perform the data analyses for this study.

## Results

### Sociodemographic Characteristics of the Study Population

The BRFSS datasets from 2017 to 2023 consisting of data from the Deep South region (AL, FL, GA, LA, MS, NC, SC, TN, and TX) comprised 476,439 respondents, representing 510,762,597 adults in the region. During the study period, 153,260 (42%) respondents reported ever been tested for HIV while 44,330 (45%) respondents reported testing for HIV in the past 12 months. The majority of respondents were NH White (55%, *n* = 312,692), 65 years or older (22%, *n* = 175,006), female (52%, *n* = 266,020), and heterosexual (93%, *n* = 161,822). Additionally, respondents were predominantly employed for wages (46%, *n* = 178,364), had some college education (31%, *n* = 130,998), married (47%, *n* = 215,392), and earned $50,000 or more annually (48%, *n* = 165,187). Table 1 provides the demographic characteristics of respondents in detail.

**Table 1:** Sociodemographic characteristics of the study population, behavioral risk factor surveillance system, 2017–2023

Characteristics	*n*	Pop. Size	%	[95% CI]
State				
Alabama Florida Georgia Louisiana Mississippi North Carolina South Carolina Tennessee Texas	39,207 92,707 57,454 35,531 35,253 33,306 63,446 37,605 81,930	27,199,062 105,544,150 57,887,737 25,012,102 15,954,436 57,980,328 28,623,069 37,872,004 154,689,709	5.3 20.7 11.3 4.9 3.1 11.4 5.6 7.4 30.3	[5.3–5.4] [20.5–20.8] [11.2–11.4] [4.9–4.9] [3.1–3.2] [11.3–11.4] [5.6–5.6] [7.4–7.5] [30.1–30.5]
**Age group**				
18–24 25–34 35–44 45–54 55–64 65 or older	28,394 51,392 58,516 72,324 90,807 175,006	62,943,723 87,645,180 84,245,518 82,345,814 82,053,293 111,529,069	12.3 17.2 16.5 16.1 16.1 21.8	[12.1–12.5] [16.9–17.4] [16.3–16.7] [16.0–16.3] [15.9–16.3] [21.6–22.0]
**Race/ethnicity**				
NH White NH Black NH Other Race NH Multiracial Hispanic	312,692 84,418 15,217 8068 44,271	274,810,498 93,956,948 22,788,955 7,764,369 99,105,561	55.1 18.9 4.6 1.6 19.9	[54.9–55.4] [18.6–19.1] [4.4–4.7] [1.5–1.6] [19.6–20.2]
**Sex**				
Male Female	210,245 266,020	246,860,783 263,713,286	48.3 51.7	[48.1–48.6] [51.4–51.9]
**Sexual orientation**				
Lesbian or Gay Heterosexual Bisexual Other	2184 161,822 4126 1917	2,730,701 159,169,260 6,183,453 2,524,356	1.6 93.3 3.6 1.5	[1.5–1.7] [93.0–93.6] [3.4–3.8] [1.4–1.6]
**Educational level**				
< High school High school Some College College Graduate	45,283 130,042 130,998 167,752	71,862,263 145,238,501 156,762,216 134,157,005	14.1 28.6 30.9 26.4	[13.9–14.4] [28.3–28.8] [30.6–31.1] [26.2–26.6]
**Income (USD)**				
<$15k $15k–<$25k $25k–<$35k $35k–<$50k $50k or more	38,645 62,220 44,851 53,033 165,187	38,185,899 63,583,478 47,318,578 54,169,027 183,656,721	9.9 16.4 12.2 14.0 47.5	[9.7–10.1] [16.2–16.7] [12.0–12.4] [13.8–14.2] [47.1–47.8]
**Employment status**				
Employed for wages Self-Employed Unemployed Retired	178,364 38,001 101,743 149,424	229,683,524 48,020,887 122,789,001 98,186,979	46.1 9.6 24.6 19.7	[45.8–46.3] [9.5–9.8] [24.4–24.9] [19.5–19.9]
**Marital status**				
Not married Married Divorced Widowed	101,510 215,392 91,193 64,104	142,728,874 239,234,875 83,671,187 39,981,780	28.2 47.3 16.5 7.9	[28.0–28.5] [47.0–47.6] [16.3–16.8] [7.8–8.0]
**Ever tested for HIV?**				
Yes No	153,260 257,073	184,943,237 251,226,188	42.4 57.6	[42.1–42.7] [57.3–57.9]
**Tested for HIV ≤ 12 months?**				
HIV testing ≤ 12 months HIV testing > 12 months	44,330 64,577	62,890,820 75,847,608	45.3 54.7	[44.8–45.9] [54.1–55.2]

n—unweighted count; Pop. Size—weighted count (population size); %—weighted percentage; CI—confidence interval, NH—non-Hispanic, NH Other race—Asians, American Indians, and Native Hawaiians, USD—United States Dollar

### Trends in Ever Been Tested for HIV Among Adults in the Deep South from 2017 to 2023

In the Deep South, the percentage of adults ever undergoing HIV testing increased from 43% in 2017 to 47% in 2019 (APC: 2.6; 95% CI: − 12.4, 20.1), then decreased to 40% in 2022, followed by a slight increase to 41% in 2023 (APC: − 3.1; 95% CI: − 8.0, 2.0). Though the overall trend indicated a decrease, it was not statistically significant (AAPC: − 1.3; 95% CI: − 4.0, 1.6). Additionally, all states in the Deep South, except LA, experienced a decrease in the percentage of ever been tested for HIV among adults. However, only NC experienced a significant decrease from 45% in 2017 to 40% in 2023 (AAPC: − 3.0; 95% CI: − 5.7, − 0.2). Findings also revealed a slight decrease in ever been tested for HIV from 36 to 31% among 18–24-year-olds (AAPC: − 4.7; 95% CI: − 9.7, 0.7), and significant decreases from 59 to 51% and 63–57% among 25–34-year-olds (AAPC: − 3.6; 95% CI: − 6.5, − 0.6) and 35–44-year-olds (AAPC: − 2.3; 95% CI: − 4.0, − 0.5), respectively, during the same period. In contrast, there were increased trends among adults aged 45 or older, although not statistically significant.

Furthermore, there were decreases in ever been tested for HIV among all racial/ethnic groups during the study period, except among NH White adults. However, a significant decrease was only observed among NH Black (AAPC:– 2.4; 95% CI: − 4.4, − 0.4) and NH multiracial adults (AAPC:– 2.9; 95% CI: − 5.5, − 0.1). Similarly, there was a significant decrease in ever been tested for HIV among respondents who identified as lesbian or gay from 72% in 2017 to 51% in 2023 (AAPC: − 6.7; 95% CI: − 10.3, − 3.0). However, the percentage decrease observed among heterosexuals, bisexual, or “other” adults was not significant. Among sexes, a decreased trend was observed in both males (AAPC: − 1.5; 95% CI: − 3.6, 0.5) and females (AAPC: − 1.5, 95% CI: − 4.2, 1.2), though this trend was not statistically significant.

The percentage of ever been tested for HIV also decreased across all education and income levels from 2017 to 2023, with a significant decrease observed only among college graduates (AAPC: − 2.1; 95% CI: − 4.0, − 0.0). Across various employment and marital status categories, a decrease in ever testing for HIV was mostly observed. However, during the same period, increased trends from 20 to 24% and 52–53% were observed among retired (AAPC: 2.0; 95% CI: − 0.8, 4.8) and divorced persons (AAPC: 3.6; 95% CI: − 3.4, 11.0), respectively, although not statistically significant.

**Fig. 1:**
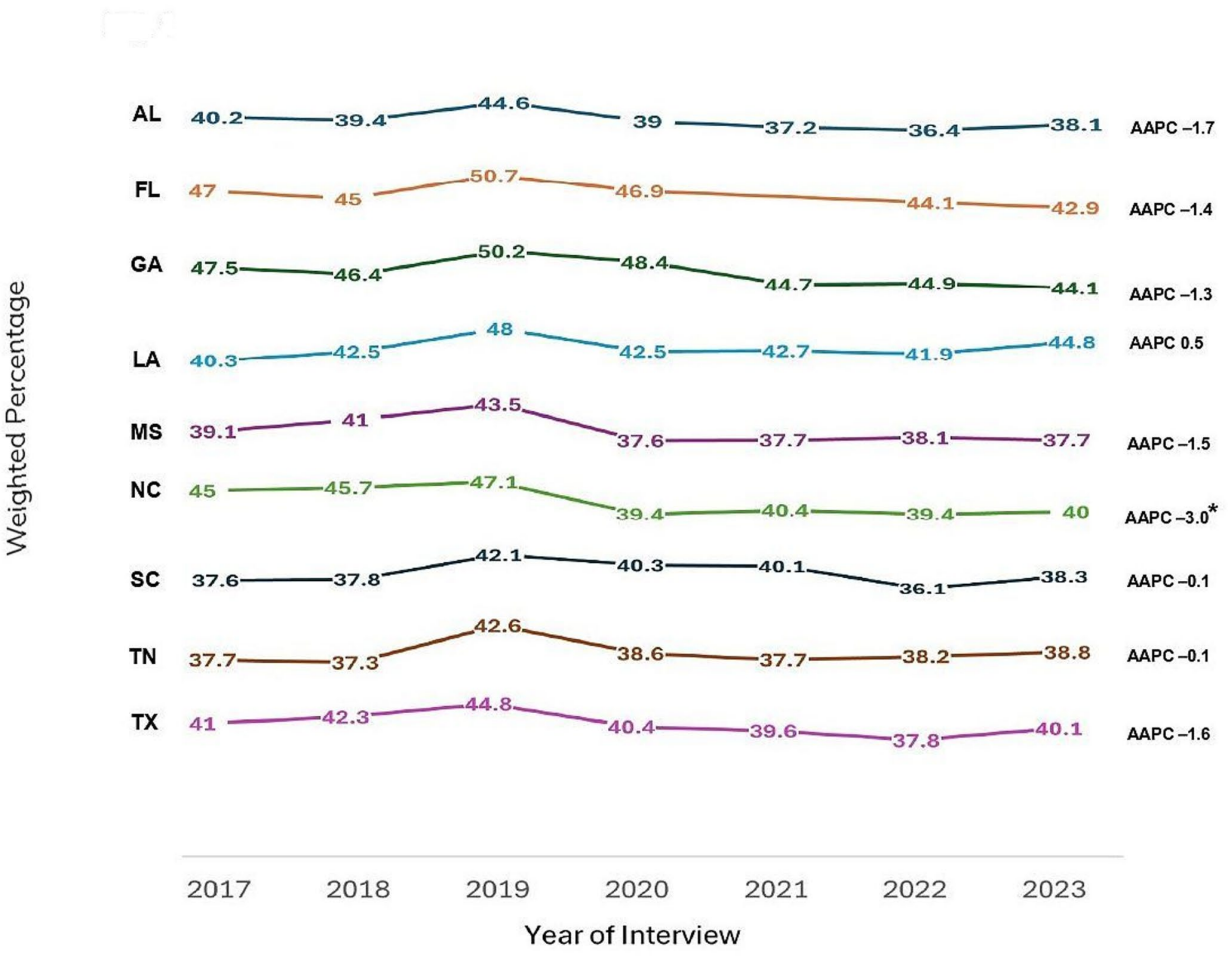
Trends in ever been tested for HIV by state among adults in the deep south, behavioral risk factor surveillance system, 2017–2023. The Deep South is defined as Alabama [AL], Florida [FL], Georgia [GA], Louisiana [LA], Mississippi [MS], North Carolina [NC], South Carolina [SC], Tennessee [TN], and Texas [TX]. *Denotes statistical significance (Average Annual Percent Change [AAPC] 95% confidence interval does not include 1)

Table 2 presents the weighted percentages of ever been tested for HIV by year of interview, along with the AAPCs and corresponding 95% CIs of each variable. Figure 1 illustrates the graphical depiction of overall trends in ever been tested for HIV by state among adults in the Deep South from 2017 to 2023.

**Table 2:** Weighted percentages and average annual percent change in ever been tested for HIV among adults in the deep South, behavioral risk factor surveillance system, 2017–2023

	2017 (*n* = 79079)		2018 (*n* = 74070)		2019 (*n* = 71181)		2020 (*n* = 63028)		2021 (*n* = 52894)		2022 (*n* = 71056)		2023 (*n* = 65131)		Total (*N* = 476439)	AAPC [95% CI]
Category	*n*	%	*n*	%	*n*	%	*n*	%	*n*	%	*n*	%	*n*	%	*N*	
**Overall**	24,817	43.0	24,560	43.0	24,087	46.7	20,553	42.5	16,604	40.2	21,398	40.2	21,241	41.0	153,260	–1.3 [–4.0, 1.6]
**State**																
Alabama Florida Georgia Louisiana Mississippi North Carolina South Carolina Tennessee Texas	2022 7994 2152 1403 1280 1776 3021 1635 3534	40.2 47.0 47.5 40.3 39.1 45.0 37.6 37.7 41.0	2060 5373 3439 1699 1984 1854 2989 1519 3643	39.4 45.0 46.4 42.5 41.0 45.7 37.8 37.3 42.3	2307 6060 2621 1688 1710 1701 2204 1885 3911	44.6 50.7 50.2 48.0 43.5 47.1 42.1 42.6 44.8	1712 4053 3260 1497 1836 1985 1281 1357 3572	39.0 46.9 48.4 42.5 37.6 39.4 40.3 38.6 40.4	1359 N/A 2666 1642 1312 1807 2779 1457 3582	37.2 N/A 44.7 42.7 37.7 40.4 40.1 37.7 39.6	1296 4125 2921 1747 1373 1559 2606 1530 4241	36.4 44.1 44.9 41.9 38.1 39.4 36.1 38.2 37.8	1386 4372 2691 1960 1319 1425 2962 1756 3370	38.1 42.9 44.1 44.8 37.7 40.0 38.3 38.8 40.1	12,142 31,977 19,750 11,636 10,814 12,107 17,842 11,139 25,853	–1.7 [–4.9, 1.6] –1.4 [–4.4, 1.8] –1.3 [–3.1, 0.6] 0.5 [–2.6, 3.7] –1.5 [–4.2, 1.2] –3.0 [–5.7, − 0.2]* − 0.1 [–2.5, 2.4] –0.1 [–2.6, 2.6] –1.6 [–4.0, 0.8]
**Age group**																
18–24 25–34 35–44 45–54 55–64 65 or older	1419 4256 4743 5271 5043 4085	36.3 59.0 63.2 52.0 35.7 16.9	1383 4175 4912 5126 4812 4152	34.9 57.8 62.2 51.1 37.4 18.6	1405 3899 4551 4947 4923 4362	40.5 62.4 65.3 56.2 42.4 20.6	1042 3230 4162 4198 3905 4016	29.8 53.3 58.8 52.1 40.3 22.9	812 2488 3448 3695 3080 3081	29.6 50.0 56.5 50.9 37.0 19.5	999 2972 4267 4496 4269 4395	26.7 48.7 57.2 53.8 40.0 20.2	1070 2974 4130 4436 4161 4470	30.6 50.5 56.6 52.6 41.0 21.6	8130 23,994 30,213 32,169 30,193 28,561	–4.7 [–9.7, 0.7] –3.6 [–6.5, − 0.6]* –2.3 [–4.0, − 0.5]* 0.1 [–1.8, 2.1] 1.4 [–1.6, 4.5] 2.8 [–1.1, 6.9]
**Race/ethnicity**																
NH White NH Black NH Other Race NH Multiracial Hispanic	14,221 5950 816 539 2725	36.2 62.4 37.2 57.4 45.0	13,592 6779 794 541 2362	35.9 63.2 36.4 59.8 44.7	13,695 5849 787 643 2638	39.2 65.9 38.3 62.0 52.0	11,374 5151 695 472 2454	35.5 61.7 31.9 56.0 44.5	9105 4455 555 438 1702	34.7 56.7 31.1 55.7 39.8	12,210 5212 535 526 2304	33.7 57.5 29.7 49.1 42.8	11,959 4854 822 572 2356	35.0 55.1 34.4 51.5 43.6	86,156 38,250 5004 3731 16,541	1.3 [–3.6, 1.1] –2.4 [–4.4, − 0.4]* –3.0 [–6.7, 0.8] –2.9 [–5.5, − 0.1]* –1.6 [–5.3, 2.3]
**Sex**																
Male Female	10,842 13,963	41.9 44.0	10,705 13,827	41.4 44.4	10,428 13,659	44.8 48.6	9183 11,370	40.8 44.0	7612 8992	39.0 41.4	9870 11,528	38.9 41.5	9823 11,418	39.9 42.0	68,463 84,757	–1.5 [–3.6, 0.5] –1.5 [–4.2, 1.2]
**Sexual orientation**																
Lesbian or Gay Heterosexual Bisexual Other	491 17,393 467 60	72.2 42.3 55.9 30.2	134 8361 363 85	57.2 42.6 63.1 41.3	182 9944 429 119	65.9 47.4 62.6 43.4	98 5189 245 104	54.1 43.4 58.7 45.2	94 4844 236 83	48.4 42.0 48.5 32.7	105 4546 265 89	49.2 41.4 44.6 35.4	114 4693 264 95	51.4 41.6 54.0 37.8	1218 54,970 2269 635	–6.7 [–10.3, 3.0]* –0.5 [–3.3, 2.3] –3.2 [–7.9, 1.7] –0.5 [–8.3, 8.0]
**Education level**																
< High School High School Some College College Grad	2501 6303 7454 8501	40.1 39.8 45.7 45.2	2394 6241 7436 8428	36.5 40.1 46.9 45.2	2457 6205 7296 8066	43.8 45.1 48.8 47.8	1980 5320 6185 7003	39.8 39.7 45.8 42.9	1367 3867 4949 6357	38.0 36.5 43.6 41.5	1550 4965 6527 8281	36.8 36.7 43.5 41.9	1606 4955 6254 8343	37.9 37.7 44.9 41.7	13,855 37,856 46,101 54,979	–1.1 [–4.3, 2.3] –2.1 [–5.3, 1.3] –1.2 [–3.0, 0.6] –2.1 [–4.0, − 0.0]*
**Annual income (USD)**																
<$15k $15k–<$25k $25k–<$35k $35k–<$50k $50k or more	3141 4599 2371 2889 8783	47.9 45.9 45.8 44.4 43.3	2846 4250 2395 2888 8985	46.8 46.6 45.6 44.6 43.5	2893 4113 2157 2720 8906	52.4 49.8 49.7 47.1 47.5	2318 3402 1894 2355 7719	47.9 45.5 46.0 44.0 43.0	1346 1831 1939 1945 6992	48.7 43.4 40.9 43.7 41.3	1572 2269 2433 2405 5105	45.3 43.3 42.8 41.7 41.1	1481 1961 2197 2503 10,007	47.1 44.4 41.6 42.1 42.9	15,597 22,425 15,386 17,705 56,497	–0.6 [–3.1, 2.0] –1.3 [–3.7, 1.3] –2.1 [–4.7, 0.5] –1.4 [–3.0, 0.2] –1.0 [–3.5, 1.5]
**Employment status**																
Employed Self-employed Unemployed Retired	11,518 2195 7062 3830	50.4 48.2 44.8 19.5	11,661 2146 6617 3917	49.6 46.0 45.3 21.6	11,001 2202 6655 4039	53.5 50.6 51.8 22.6	9394 1756 5613 3618	47.4 47.2 45.7 24.3	7999 1485 4099 2880	44.7 45.5 43.6 22.1	10,132 2028 4957 4056	45.9 45.1 43.4 22.2	9948 2018 4964 4073	45.9 47.7 44.8 23.5	71,653 13,830 39,967 26,413	–2.2 [–4.5, 0.2] –0.7 [–2.7, 1.3] –1.0 [–4.4, 2.5] 2.0 [–0.8, 4.8]
**Marital status**																
Not married Married Divorced Widowed	7344 10,998 4636 1702	50.6 39.2 51.9 22.6	1632 7635 10,616 4519	22.1 50.1 39.0 53.5	7365 10,350 4373 1820	55.2 42.7 55.6 25.1	6166 8975 3692 1581	46.6 39.6 53.8 27.5	4926 7536 2816 1180	44.3 37.3 52.5 25.0	6232 9782 3707 1492	43.6 37.7 53.9 22.5	6315 9607 3607 1547	44.6 38.4 53.2 26.0	39,980 64,883 33,447 13,841	–2.6 [–9.4, 4.8] –2.7 [–6.8, 1.7] 3.6 [–3.4, 11.0] –11.8 [27.6, 7.3]

Acronym: n—unweighted count, N—total unweighted count, %—weighted percentage, AAPC—Average Annual Percentage Change, CI—confidence interval, NH—non-Hispanic, NH Other race—Asians, American Indians, and Native Hawaiians, N/A—data not available, USD—United States Dollar, *—significant finding

### Trends in HIV Testing in the Past 12 Months Among Adults in the Deep South from 2017 to 2023

The percentage of testing for HIV in the past 12 months among adults decreased from 48% in 2017 to 47% in 2019 (APC: − 1.6; 95% CI: − 9.8, 7.4), followed by a further decrease to 42% in 2022 and a slight increase to 43% in 2023 (APC: − 2.8; 95% CI: − 6.0, 0.6), with the overall decline being significant (AAPC: − 2.4; 95% CI: − 4.0, − 0.7). Additionally, all states in the Deep South experienced a decrease in HIV testing within the past 12 months, except for AL which experienced a slight increase (AAPC: 0.1; 95% CI: − 2.5, 2.8). However, significant declines were observed only in FL (AAPC: − 3.5; 95% CI: − 4.8, − 2.2), GA (AAPC: − 4.3; 95% CI: − 6.2, − 2.5), LA (AAPC: − 2.6; 95% CI: − 4.5, − 0.7), NC (AAPC: − 2.8; 95% CI: − 4.8, − 0.9), and SC (AAPC: − 4.0, 95% CI: − 6.3, − 1.7).

Across age groups, there were significant declines in HIV testing within the past 12 months from 2017 to 2023, except among individuals aged 35–44 (AAPC: − 0.7; 95% CI: − 1.2, 2.6). There were also significant decreases among NH White and NH Black individuals from 38 to 34% (AAPC: − 3.0; 95% CI: − 4.3, − 1.7) and 66–54% (AAPC: − 3.5, 95% CI: − 4.3, − 2.7), respectively. Additionally, there were decreases among other racial groups, although not statistically significant.

Furthermore, from 2017 to 2023, there were significant decreases in HIV testing in the past 12 months among males (AAPC: − 2.6; 95% CI: − 4.0, − 1.2) and females (AAPC: − 2.2; 95% CI: − 3.0, − 1.5). Decreased trends were also observed across all sexual identities; however, a significant decrease was only observed among heterosexual adults from 47% in 2017 to 39% in 2023 (AAPC: − 2.6; 95% CI: − 3.9, − 1.2).

Among education levels, the only significant decline was among adults with some college education, with testing rates decreasing from 51% in 2017 to 44% in 2023 (AAPC: − 3.0; 95% CI: − 4.7, − 1.3). Additionally, there were significant decreases observed among respondents earning an annual income of less than $35,000 compared to their counterparts earning higher. Significant decreases were also observed across all employment categories except for respondents who were self-employed, where the decrease observed was not significant (AAPC: − 0.9; 95% CI: − 4.6, 2.9). Among marital status categories, decreased trends in HIV testing within the past 12 months were observed; however, these decreases were not statistically significant.

**Fig. 2:**
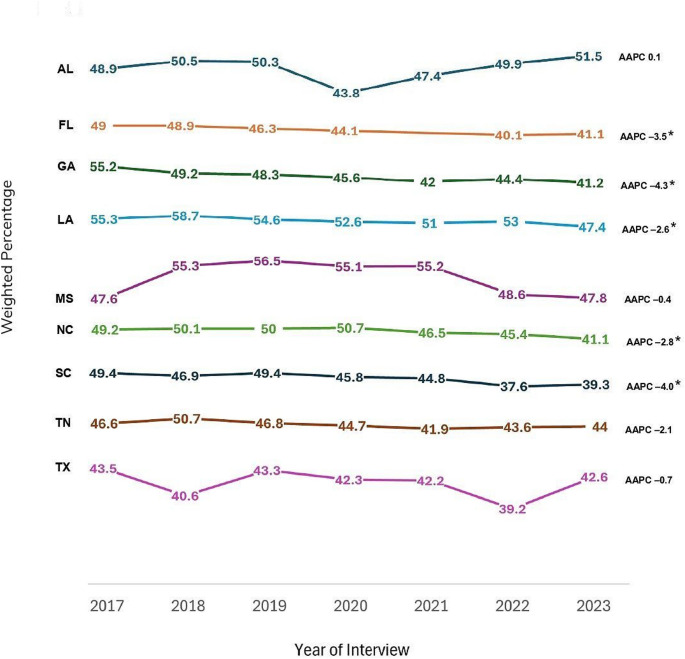
Trends in HIV testing in the past 12 months by state among adults in the deep south, behavioral risk factor surveillance system, 2017–2023. The Deep South is defined as Alabama [AL], Florida [FL], Georgia [GA], Louisiana [LA], Mississippi [MS], North Carolina [NC], South Carolina [SC], Tennessee [TN], and Texas [TX]. *Denotes statistical significance (Average Annual Percent Change [AAPC] 95% confidence interval does not include 1)

**Fig. 3:**
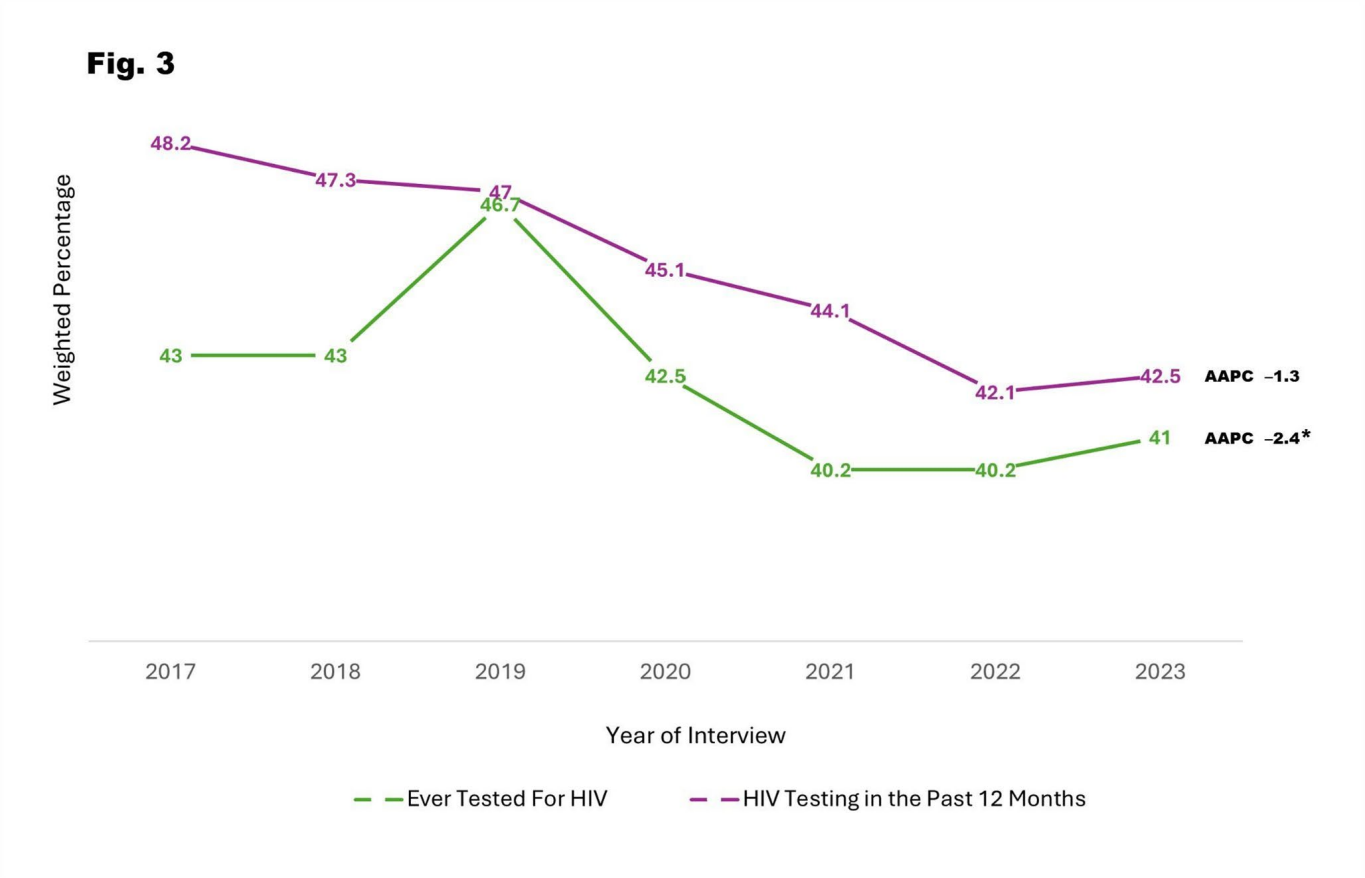
Trends in HIV testing among adults in the deep south: behavioral risk factor surveillance system, 2017–2023. The Deep South is defined as Alabama [AL], Florida [FL], Georgia [GA], Louisiana [LA], Mississippi [MS], North Carolina [NC], South Carolina [SC], Tennessee [TN], and Texas [TX]. *Denotes statistical significance (Average Annual Percent Change [AAPC] 95% confidence interval does not include 1)

Table 3 presents the weighted percentages of HIV testing in the past 12 months by year of interview, along with the AAPCs and corresponding 95% CIs of each variable. Figure 2 presents a graphical depiction of overall trends in HIV testing in the past 12 months by state among adults in the Deep South between 2017 and 2023. Additionally, Fig. 3 visually presents overall trends in ever been tested for HIV and HIV testing in the past 12 months among adults in the Deep South between 2017 and 2023.

**Table 3:** Weighted percentages and average annual percent change in HIV testing in the past 12 months among adults in the deep South, behavioral risk factor surveillance system, 2017–2023

	2017 (*n* = 79079)		2018 (*n* = 74070)		2019 (*n* = 71181)		2020 (*n* = 63028)		2021 (*n* = 52894)		2022 (*n* = 71056)		2023 (*n* = 65131)		Total (*N* = 476439)	AAPC [95% CI]
Category	*n*	%	*n*	%	*n*	%	*n*	%	*n*	%	*n*	%	*n*	%	*N*	
**Overall**	8002	48.2	7647	47.3	7607	47.0	5700	45.1	4462	44.1	5429	42.1	5483	42.5	44,330	–2.4 [–4.0, − 0.7]*
**State**																
Alabama Florida Georgia Louisiana Mississippi North Carolina South Carolina Tennessee Texas	625 2496 815 508 426 596 996 464 1076	48.9 49.0 55.2 55.3 47.6 49.2 49.4 46.6 43.5	626 1637 1169 620 631 566 889 443 1066	50.5 48.9 49.2 58.7 55.3 50.1 46.9 50.7 40.6	686 1848 978 637 554 533 657 526 1188	50.3 46.3 48.3 54.6 56.5 50.0 49.4 46.8 43.3	408 1052 979 454 530 558 337 340 1042	43.8 44.1 45.6 52.6 55.1 50.7 45.8 44.7 42.3	336 N/A 718 475 375 472 719 341 1026	47.4 N/A 42.0 51.0 55.2 46.5 44.8 41.9 42.2	322 992 791 503 370 384 594 356 1117	49.9 40.1 44.4 53.0 48.6 45.4 37.6 43.6 39.2	384 1018 758 543 395 329 703 427 926	51.5 41.1 41.2 47.4 47.8 41.1 39.3 44.0 42.6	3387 9043 6208 3740 3281 3438 4895 2897 7441	0.1 [–2.5, 2.8] –3.5 [–4.8, − 2.2]* –4.3 [–6.2, − 2.5]* –2.6 [–4.5, − 0.7]* –0.4 [–4.2, 3.5] –2.8 [–4.8, − 0.9]* –4.0 [–6.3, − 1.7]* –2.1 [–4.3, 0.2] –0.7 [–2.6, 1.2]
**Age group**																
18–24 25–34 35–44 45–54 55–64 65 or older	946 2079 1642 1417 1161 757	78.2 55.7 42.0 38.3 36.1 38.7	887 2045 1644 1354 1049 668	77.4 57.4 41.1 35.3 36.3 31.7	959 1901 1593 1262 1101 791	76.6 55.2 44.0 33.9 35.1 33.7	652 1479 1331 938 711 589	71.8 54.6 45.3 33.2 30.2 33.2	473 1086 1047 884 547 425	66.7 54.5 40.7 35.3 30.9 32.0	554 1261 1322 943 734 615	67.4 50.4 44.8 31.4 30.3 28.7	591 1272 1326 1005 711 578	68.3 52.4 43.3 31.7 31.7 25.2	5062 11,123 9905 7803 6014 4423	–2.9 [–4.1, − 1.6]* –1.6 [–2.8, − 0.3]* –0.7 [–1.2, 2.6] –2.7 [–4.6, − 0.8]* –3.2 [–5.3, − 1.0]* –5.1 [–8.3, − 1.8]*
**Race/ethnicity**																
NH White NH Black NH Other Race NH Multiracial Hispanic	3562 2745 271 195 1058	38.4 65.5 50.6 46.7 47.8	3303 2894 270 202 851	37.7 64.1 41.6 54.4 45.9	3432 2524 298 231 958	37.1 60.4 52.4 46.9 51.1	2436 1962 208 166 830	35.0 59.9 43.9 47.4 46.3	1887 1596 175 148 566	33.4 57.6 47.0 53.3 47.0	2412 1760 163 169 776	32.0 53.3 49.5 43.4 47.6	2452 1679 207 170 781	33.6 53.9 43.9 51.0 45.2	19,484 15,160 1592 1281 5820	–3.0 [–4.3, − 1.7]* –3.5 [–4.3, − 2.7]* –0.6 [–5.2, 4.3] –0.3 [–4.6, 4.2] –0.6 [–2.6, 1.5]
**Sex**																
Male Female	3645 4354	48.7 47.7	3537 4101	49.3 45.5	3471 4136	48.6 45.6	2620 3080	45.5 44.8	2037 2425	43.5 44.6	2562 2867	42.4 41.9	2607 2876	44.0 41.2	20,479 23,839	–2.6 [–4.0, − 1.2]* –2.2 [–3.0, − 1.5]*
**Sexual orientation**																
Lesbian or Gay Heterosexual Bisexual Other	226 5367 220 25	60.7 46.6 58.7 71.3	46 2347 158 25	44.7 43.4 56.1 29.8	62 2873 205 47	50.1 42.3 57.4 62.4	28 1424 114 40	40.6 44.1 61.7 54.4	33 1293 104 23	55.3 42.7 56.1 40.8	32 1121 108 23	48.0 40.0 52.5 47.5	29 1160 103 33	59.6 39.2 52.6 39.8	456 15,585 1012 216	–2.5 [–7.9, 3.3] –2.6 [–3.9, − 1.2]* –1.6 [–3.9, 0.7] –7.1 [–17.5, 4.7]
**Education level**																
< High School High School Some College College Grad	876 2231 2493 2389	52.3 50.0 51.3 40.6	705 2110 2445 2373	49.4 51.7 49.3 39.7	775 2134 2374 2308	53.4 51.2 45.9 41.4	530 1543 1744 1863	43.3 47.9 44.6 43.9	334 1162 1338 1617	41.7 49.1 44.7 39.9	398 1363 1663 1988	47.9 46.4 41.3 37.3	343 1396 1669 2057	35.5 48.5 44.2 37.8	3961 11,939 13,726 14,595	–4.4 [–8.7, 0.2] –1.3 [–2.7, 0.1] –3.0 [–4.7, − 1.3]* –1.3 [–3.7, 1.1]
**Annual income (USD)**																
<$15k $15k–<$25k $25k–<$35k $35k–<$50k $50k or more	1167 1742 875 955 2319	57.2 53.9 56.8 48.3 38.5	1023 1551 798 969 2418	58.3 53.2 49.6 48.8 40.0	1060 1466 736 917 2441	55.1 53.1 49.2 46.2 40.9	747 1050 571 663 1970	51.2 49.9 46.3 44.1 41.1	416 546 572 576 1767	48.5 50.4 47.1 49.1 39.0	439 656 693 654 1326	50.5 47.3 46.1 43.4 42.2	441 541 663 725 2456	51.2 42.0 45.7 48.2 39.7	5293 7552 4908 5459 14,697	–2.7 [–4.5, − 0.9]* –3.0 [–4.6, − 1.4]* –3.2 [–5.2, − 1.2]* –0.7 [–3.1, 1.8] 0.5 [–1.0, 2.0]
**Employment status**																
Employed Self-employed Unemployed Retired	4007 629 2492 786	47.7 40.8 53.1 41.6	4056 598 2212 697	48.8 38.8 50.6 35.0	3811 662 2303 760	48.1 39.3 50.0 34.4	2908 464 1716 555	45.9 46.2 47.0 31.0	2406 361 1211 439	44.8 36.4 48.9 32.1	2844 495 1414 612	42.1 37.3 48.0 28.7	2924 508 1403 584	44.5 39.3 45.3 28.4	22,956 3717 12,751 4433	–2.1 [–3.5, − 0.6]* –0.9 [–4.6, 2.9] –2.2 [–3.3, − 1.1]* –5.8 [–8.0, − 3.5]*
**Marital status**																
Not married Married Divorced Widowed	3447 2651 1445 421	61.3 37.4 48.4 41.2	347 3461 2433 1359	39.6 61.9 35.4 44.7	3448 2399 1318 387	61.5 35.3 44.9 36.7	2628 1818 931 277	60.2 34.5 40.3 30.0	1949 1549 712 205	56.7 34.2 42.1 39.9	2354 1890 877 256	54.5 32.7 40.1 36.2	2486 1828 882 242	56.0 31.5 42.4 32.8	16,659 15,596 8598 3147	–1.8 [–4.8, 1.3] –8.7 [–19.5, 3.6] –0.3 [–6.3, 6.0] –5.2 [–11.0, 1.1]

Acronym: n—unweighted count, N—total unweighted count, %—weighted percentage, AAPC—Average Annual Percentage Change, CI—confidence interval, NH—non-Hispanic, NH Other race—Asians, American Indians, and Native Hawaiians, N/A—data not available, USD—United States Dollar, *—significant finding

## Discussion

This study examined trends in HIV testing among adults in the Deep South using 2017–2023 BRFSS data. Overall, the results point to decreasing HIV testing rates across Deep South states and sociodemographic factors. Despite ongoing public health efforts by the CDC to prevent and diagnose new HIV infections early [31], these decreasing trends indicate challenges in achieving optimal HIV testing rates in the Deep South region.

Our findings suggest that the overall percentage of ever been tested for HIV among respondents declined over the study period, however, this decrease was not statistically significant. Our results also suggest an overall significant decrease in the percentage of HIV testing in the past 12 months among adults, implying that fewer adults are being tested for HIV, either routinely or at any time, during the past year. Annual testing is imperative for the prevention and early diagnosis of HIV, particularly in a region such as the Deep South where the HIV prevalence is high [8].

Prior to our study period, national trends highlighted both an increase and decrease in HIV testing prevalence. According to the CDC [32], the National Health and Nutrition Examination Survey (NHANES) showed the percentage of adults who had ever been tested for HIV decreased from 42.5% in the 1999–2000 survey to 38.1% in the 2001–2002 survey, before rising again to 43.1% in the 2009–2010 survey. Additionally, the National Health Interview Survey data showed that the percentage of adults who had ever been tested for HIV increased significantly from 36.6% in 2000 to 45.0% in 2010, though trends fluctuated over time based on NHANES data for the same period [32]. Furthermore, between 2016 and 2017, Pitasi et al. [33] reported that 38.9% of U.S. adults aged ≥ 18 years had ever been tested for HIV, with only 29.2% of individuals at higher risk for HIV being tested in the past year. Pitasi et al. [33] attributes these suboptimal testing rates to missed opportunities to fully implement HIV screening recommendations and the variability in testing practices across jurisdictions. In the South specifically, studies highlight lack of awareness of HIV status and non-expansion of Medicaid as reasons for suboptimal HIV testing rates in this region [34, 35].

Our results also identified subpopulations that experienced significant declines in testing rates, illustrating potential disparities in HIV testing. For example, the percentage of ever been tested for HIV decreased significantly in North Carolina, among adults who were 25–44 years old, NH Black, NH multiracial, and identifying as lesbian or gay. These trends are particularly concerning given that studies show that certain populations including younger adults, Blacks/African Americans, and sexual minorities are at an increased risk of contracting HIV [2, 36, 37]. For instance, HIV.gov [2] highlighted that individuals aged 13 to 34 made up 60% of the estimated new HIV infections in 2022. Additionally, the CDC [38] noted that in 2021, Black people represented 12% of the U.S. population but comprised 40% of the estimated 32,100 new HIV infections that year. In the same year, Black gay and bisexual men were the most impacted group, comprising 37% of the estimated new infections among all gay and bisexual men [36]. In 2022, men who had sex with men made up 67% of new HIV diagnoses across the United States, including six territories and freely associated states [38]. Yet, our findings reveal a significant decrease in HIV testing among these at-risk populations, which could be attributed to several barriers such as limited access to healthcare, stigma, confidentiality concerns, negative treatment by healthcare staff, low perceived risk, and disruptions from the COVID-19 pandemic, among others [16, 34].

Furthermore, results showed a significant decrease in HIV testing within the past 12 months in FL, GA, LA, NC, and SC, as well as among various demographic groups, from 2017 to 2023. For example, there was a significant decline in testing within the past 12 months across all adult age groups except 35–44, as well as among NH White, NH Black, male, female, and heterosexual adults, among others. These findings suggest that declines in recent HIV testing are not confined to any single group but are widespread across different demographics. The decrease among males and females, and once again, among non-Hispanic Black adults, is particularly concerning given the disproportionate burden of HIV in these communities [39, 40]. The Kaiser Family Foundation [41] revealed that the rate per 100,000 of new HIV diagnosis among Black adults and adolescents (41.6) was approximately eight times higher than that of White individuals (5.3) and double the rate for Latinos (23.4) in 2022. Additionally, Black men had the highest rate across all racial/ethnic and gender groups at 66.3, while Black women (19.2) had the highest rate among women [41].

The COVID-19 pandemic may have played a role in the overall decline of HIV testing rates during this period, as healthcare systems were overwhelmed, and many non-emergency services, including HIV testing, were disrupted [42]. Additionally, several shelter-in-place orders across the U.S., beginning as early as March 2020, restricted the movement of persons from place-to-place. Moreover, the fear of exposure to COVID-19 in healthcare settings may have further reduced access to testing during this period, exacerbating the decline. Nosyk et al. [43] reported that HIV testing in the United States dropped significantly due to the COVID-19 pandemic, with the CDC recording 1,338,665 tests in 2020—a 44% decrease from 2019 and a 56% decrease from 2015, when 3,026,074 tests were conducted under CDC funding. Similarly, DiNenno et al. [44] noted that the pandemic disrupted healthcare services, leading to a 17% reduction in new HIV diagnoses reported to the CDC from 2019 to 2020, alongside a notable decline in HIV testing, including among priority populations within CDC-funded jurisdictions.

Despite these downward trends, we noted some positive trends during the study period. For example, in Alabama, there was a slight increase in HIV testing within the past 12 months, although there was a slight decreased trend in ever been tested for HIV in the state. Similarly, among adults earning an annual income of $50,000 or more, there was a slight decline in the rate of ever having been tested for HIV, but a slight increase in the percentage of those who tested in the past 12 months. These findings suggest that while fewer individuals in certain groups may have ever been tested for HIV, recent testing initiatives may have had some success in encouraging those already aware of their risk to continue regular testing.

While several trends in HIV testing rates among adults in the Deep South from 2017 to 2023 indicate significant declines, several other trends were not statistically significant. For instance, both males and females and Hispanic adults exhibited decreasing trends in ever having been tested for HIV, while non-Hispanic White adults showed an increase, though these trends were not significant. In terms of HIV testing within the past 12 months, non-significant declines were observed among adults aged 35–44, self-employed individuals, and those earning $35,000 or more, just to name a few. The lack of statistical significance in these trends may be attributed to factors such as variations in healthcare access, shifts in public health priorities, or differences in risk perception across subpopulations [34]. These findings may also suggest that testing rates may be stagnating or slightly declining among some subpopulations, which could have long-term implications for HIV prevention efforts.

These mixed results in HIV testing rates underscore the complexity of HIV testing behaviors. Similar to our findings, Ansa et al. [20], using BRFSS data from Georgia (2011–2015), reported slight declines in ever testing for HIV among young adults aged 18–24, as well as among males and females. Patel et al. [21], utilizing national BRFSS data from 2011 to 2017, also observed significant declines in HIV testing within the past 12 months among adults aged 34 years or younger. In contrast to our findings, Patel et al. [21] reported significant increases in ever testing for HIV and HIV testing within the past 12 months overall, as well as significant increases in ever testing for HIV among NH White, Hispanic, male, and female adults.

The significant decreases in testing among certain high-risk groups, such as young adults and racial and sexual minorities, highlight the urgency for tailored public health initiatives that specifically address the barriers faced by these populations such as limited access to healthcare, stigma, and mistrust, among others [16, 34]. Meanwhile, the observed increases in testing in Alabama, for example, may suggest that localized efforts may be effective in some contexts. Additionally, increased access to PrEP may have positively influenced HIV testing patterns, as PrEP uptake and ongoing PrEP use necessitates routine testing—every three months for oral PrEP and every two months for injectable PrEP, according to CDC guidelines [12, 45]. Public health programs should focus on reducing barriers to testing, including stigma and limited healthcare access, expanding HIV testing and PrEP programs and access, while also tailoring interventions to meet the specific needs of the most affected communities. This might include expanding community-based testing, increasing PrEP and testing awareness campaigns, and addressing the underlying social determinants of health, such as poverty and lack of insurance [46], which could help improve access to testing services and reduce the disparities.

### Strengths and Limitations

The strengths of this study include the large, diverse sample size from the BRFSS and its sampling design, which supports generalizability of the findings. Additionally, the use of weighted data allows for robust statistical analysis that accounts for sampling variability and population differences. However, BRFSS relies on self-reported data, which may overestimate or underestimate HIV testing trends. Additionally, self-reported data may be subject to recall bias, which may have contributed to the observed higher percentage of HIV testing in the past 12 months compared to the percentage of individuals who have ever been tested for HIV. Individuals who were tested within the past year may be more likely to recall and report their testing behavior, whereas those tested further in the past may underreport their testing history. Another limitation of the BRFSS data is its inability to capture contextual factors that may play a role in testing behaviors, such as stigma, or public health policies that may vary by state. Lastly, this study did not adjust for potential confounders, including health insurance coverage, which may have influenced HIV testing behaviors. Future research should consider incorporating health insurance data to better account for its potential impact on testing trends.

## Conclusion

While there have been modest gains in HIV testing in certain areas and among some groups, the overall decline in testing rates, particularly among high-risk populations, calls for scaling HIV prevention and care using mobile clinics, outreach testing programs, and free testing programs to eliminate disparities in HIV prevention and care. Given that declines in HIV testing were largely observed during the COVID-19 pandemic, these trends should be revisited now that we are in the post-pandemic period.

HIV testing is a critical entry point to both HIV prevention and care, aligning with the DHHS’s commitment under the EHE plan to diagnose all individuals with HIV as early as possible [47]. Our findings highlight the need for targeted interventions to sustain and enhance HIV testing efforts in the Deep South.
